# Coupling pH-Responsive Dyes to Agarose Hydrogels for Monitoring Metabolic States of Encapsulated Phototrophic Microbial Consortia

**DOI:** 10.1021/acsabm.5c01556

**Published:** 2025-11-12

**Authors:** Matthias Ueberham, Christian Danneberg, Lisa-Maria Wagner, Tilo Pompe

**Affiliations:** Institute of Biochemistry, 9180 Leipzig University, Leipzig 04103, Germany

**Keywords:** pH sensor, hydrogel, microbial consortia, agarose, biofilm

## Abstract

The interaction of microbial consortia within biofilms leads to emergent properties such as high resistance to environmental fluctuations, efficient biocatalytic performance, and stable metabolic states. However, the mechanisms governing these interactions are hard to capture and are not fully understood. Agarose has proven to be a good mimic of the extracellular polymeric substance, which is the polymeric matrix supporting the connectivity of microbial consortia within biofilms. We aimed at modifying polymeric agarose to generate *in situ* sensing functionalities for monitoring metabolic states within the encapsulated microbial consortia. For that, agarose was end-on chemically modified to couple the pH-responsive FAM dye. After reconstitution of hydrogels out of the functionalized agarose, a stable covalent coupling of the dyes was demonstrated using fluorescence recovery after photobleaching, showing a reduction of free FAM dye from 51% using common water washing procedures to no measurable amount of free dye using our washing procedure. Furthermore, the ability to monitor relevant pH ranges between 6 and 8 was experimentally demonstrated in the hydrogels by laser scanning microscopy. Furthermore, the application of the functional agarose hydrogels was shown in cell cultures with chemoheterotrophic and phototrophic microbial strains (*Pseudomonas taiwanensis* and *Synechocystis sp.*). The monitoring of pH changes of microbial consortia dependent on their metabolic performance over up to 3 days was proven, paving the road to the utilization of such functional agarose matrices to study metabolic interactions in complex microbial consortia.

## Graphical abstract

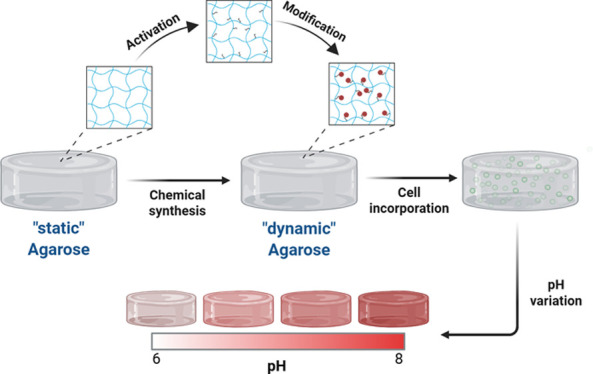

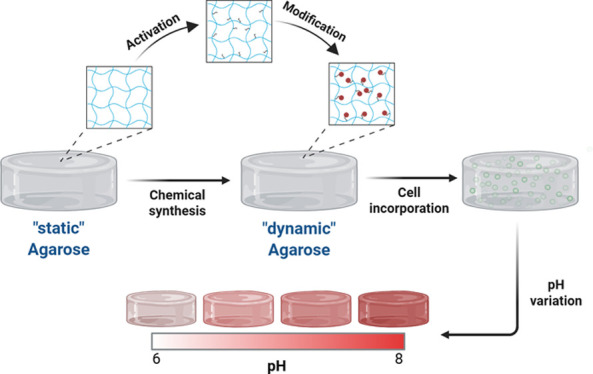

## 1 Introduction

In nature, microbial cells can form aggregated, structured communities called ‘biofilms’. Due to the high cell density, numerous intercellular interactions are enabled within biofilms leading to new, emergent properties, which are not observed in low-density, liquid culture.^1^ Many of these emergent properties, like high robustness, high biocatalyst density, and shared reaction cascades in locally structured systems, make biofilms highly attractive for industrial applications like clearance of wastewater, filtration of drinking water, and production of chemicals and biofuels. Furthermore, the built-in sustainability makes them highly interesting technological targets for future industrial process, as they are established and functional at low temperature and low pressure, can use natural energy sources like sun light, and exhibit carbon-capture abilities as in phototrophic microbial systems.^2^

However, the huge technological potential of biofilms is not yet exploited because of a limited understanding of the interactions and metabolic states of microbial consortia within biofilms and a limited availability of engineering tools to implement biofilm features in industrial processes. Therefore, material engineering approaches are envisioned to construct biofilm mimics for the investigation of principles governing microbial cell behavior in biofilms. This will eventually enable a transfer of gained knowledge into engineering tools to establish new biocatalytic processes for sustainable energy and chemical production.^3^

Within biofilms, the interplay of different microbial cells and their self-organization into cell compartments frequently leads to spatially highly structured and heterogeneous microbial consortia with different metabolic and catabolic states. It is of utmost importance to receive knowledge and control of these heterogeneous states with high spatial resolution for engineering biomimetic systems in the lab and in biotechnological applications. As is known from naturally occurring structured biofilm systems, like the microbial mats, local pH value varies a lot within the structured consortia.^4–6^ The pH value is not only indicative of the metabolic conditions of the cells but strongly influences catabolic performance of the cells in various (bio)­chemical reactions. Therefore, investigation of pH within microbial consortia, such as biofilms, is an advantageous approach to determine the metabolic state of cells.^7,8^

Microbial cells in biofilms are enclosed in a self-produced matrix called the extracellular polymeric substance (EPS) or the extracellular matrix (ECM). This matrix mainly consists of a polysaccharide network with a very high water content, making it comparable to a polymeric hydrogel. This is why polysaccharide-based hydrogels are considered to be particularly good ECM mimics for biofilm engineering. One prominent example of such polysaccharides is agarose, which is already frequently used in microbial cell cultures due to its nontoxic properties and its ability to allow the attachment of microbial cells, also commonly used as main component of the growth support for microbial cells of so-called agar plates.^9^

Agarose is a neutral gelling heteropolysaccharide derived from red algae, specifically the cell walls of seaweeds belonging to the *Rhodophyceae* class like *Gelidium* or *Gracilaria*. It is a linear polymer composed of β-D-galactose and 3,6-anhydro-α-L-galactose repeat units.^10,11^ Unlike most other polysaccharides, agarose contains both α-and β-glycosidic bonds and can only be hydrolyzed by the enzymes α-agarase (E.C. 3.2.1.158) and β-agarase (E.C. 3.2.1.81), which only few microorganisms possess, making it quite stable against degradation. Due to being extracted from natural sources, commercially available agarose’s properties can vary. However, it typically exhibits a high degree of polymerization with around 800 sugar residues, leading to a molecular weight of above 120 kDa and a sulfate content of less than 0.15%.^11^

In terms of hydrogel formation, agarose is known to yield tough, nanoporous hydrogels that can form in physiological conditions.^12^ As a physically cross-linked hydrogel, its gelation mechanism is completely governed by hydrogen bonding, by bond formation between agarose molecules as well as stabilization by structured water.^13,14^ Agarose gelation is considered as a two-step process: (1) the formation of double-helical associates from single chains and (2) a subsequent further aggregation into three-dimensional networks.^15,16^ These steps involve coaxial single and double helices formation, which are triggered by interchain interactions of hydroxyl groups and interactions with water.^11^ As hydrogen bond formation in water is well-known to be disturbed by chaotropic agents and proton scavengers, this influence is also known for agarose gelling.^11^ As a physically cross-linked hydrogel, agarose hydrogel formation is highly entropy-dependent, leading to thermally reversible gel formation.^17^ Agarose hydrogels can be solubilized in water above the agarose melting temperature (∼80–90 °C). Gel formation upon cooling is a typical phase transition process occurring above a critical concentration.^18–20^ The thermally reversible gelation follows a typical hysteresis, which leads to a significantly lower gelling temperature in the range of 36 °C. Chemical modification of the agarose polymer backbone decreases the interaction strength between the polymer chains during gelation, resulting in a shift in melting and gelation temperature to lower values below 65 and 30 °C, respectively. This form of agarose is also called low-melting agarose, with alkylated agarose being the most common one on the market.^21^

To tailor the agarose hydrogels for chemical coupling, several published protocols exist for chemical modification of the agarose polymer backbone, including an activation with epoxide, primary amine, or carboxy groups.^11^ Since the agarose gelation mechanism is governed by hydrogen bonding of hydroxyl groups and backbone structure formation, it is not surprising that chemical modification of the agarose polymer chains influences its physical properties and agarose hydrogel formation.^9^ Studies have shown that carboxylation of the agarose backbone at the primary alcohol residue in the C6 position leads to softer hydrogels and lower gelation temperatures, compared to native agarose. These observations have been linked to disruptions in helix formation and stabilization of agarose polymer chains, leading to an altered network topology of the hydrogels.^17,22^ Furthermore, high degrees of functionalization have been shown to compromise agarose’s hydrophilic, nontoxic character.^11^

Due to the inherent properties of fluorescent dyes, namely, the shift of absorption and emission bands by the polarity of the local environment, they are frequently considered as interesting tools for optical pH measurements.^23^ Applications have to consider spectral range, pH range, and chemical and optical stability, as well as chemical moieties for coupling; hence, a broad range of different dyes exists. Frequently used examples comprise fluorescein isothiocyanate (FITC) (excitation: 450/498, emission: 520, pH range dependence: 5.50–7.50, coupling group: isothiocyanate), 2’,7’-difluorofluorescein (Oregon Green) (excitation: 450/498, emission: 520, pH range dependence: 3.75–5.50, coupling group: isothiocyanate),^24^ or seminaphthofluorescein (SNAFL) dyes like carboxy SNAFL-2 (excitation: 488, emission: 613, pH range dependence: 6.00–8.00, coupling group: succinimidyl ester).^25^

As an interesting dye in a relevant pH range, a pH dependency was previously shown for the free^26^ as well as for oligonucleotide-bound FAM.^27–29^ The pH responsiveness of the FAM dye arises from the existence of different forms of the dye at different pH values due to the presence of xanthene and benzoic acid moieties in the fluorophore. Different protonation degrees of those groups led to different ionic charge distributions within the molecule. Furthermore, the lactone formation of the benzoate carboxy group at specific pH values leads to structural analogues.^30^ At each pH value, one form of fluorophore predominates. At pH values below 3, the cationic form, with a maximum absorption at 437 nm, is predominant. At pH values between 4 and 7, the neutral form of the molecule is the most abundant. FAM samples at this pH range also possess an absorption peak at around 437 nm but have the lowest extinction due to the additional formation of a lactone, which does not absorb light in the visible range. At the pH range between 7 and 8, the monoanionic form of the dye predominates, whereas above pH 8 the dianionic form is mostly present. The dianion of the fluorescein dye not only has the highest fluorescence quantum yield but also possesses a different absorption wavelength. Whereas the monoanionic form has its absorption maxima at 437 and 475 nm, the dianionic forms absorption maximum is shifted to 490 nm.^31^ Therefore, an acidification of a dye solution progressively leads to a decrease in fluorescence extinction at 490 nm because of the blue-shift between the dianionic and the monoanionic and neutral form with a strong decrease around pH 6.^23^ Hence, pH-dependent fluorescence intensity measurements at 490 nm excitation can be used to correlate fluorescence intensity with pH value, with an increase in fluorescence intensity as pH rises.^26^

This study focuses on covalently coupling a pH-responsive dye to agarose with options to encapsulate microbial cells within dye-labeled agarose hydrogels. Focus was set on preserving agarose hydrogel formation abilities by an end-on agarose chain modification strategy while ensuring the covalent linkage of the dye molecule to the agarose hydrogel. While other recent studies showed successful production of pH-responsive agarose hydrogels, they often exhibit changing mechanical properties, inhibiting stable cell culturing inside the gel and are lacking a fast optical redout option.^32^ Using our platform, an optical readout of pH changes inside the hydrogel can be observed with options of more precise spatial resolution. Proof-of-concept experiments with encapsulated phototrophic microbial consortia demonstrate the functionality and applicability of the engineered material system as a synthetic scaffold for studying microbial behavior.

## 2 Experimental Section

### 2.1 Materials and Methods

Agarose used in this study was purchased from Sigma-Aldrich (SIGMA LifeScience, Bioreagent, low EE, Lot: SLBQ6523 V). Low-melt agarose (LM-agarose) was purchased from Carl-Roth GmbH (ROTIGarose) and specifically named as such. Aminooxy-5(6)-FAM and CF633 Dye aminooxy were obtained from Biotium. Aniline and sodium (meta)­periodate (NaIO_4_) were purchased from Sigma-Aldrich. Water used was purified by a Milli-Q water purification system. 0.1 M acetate buffer was prepared by dissolving 405.9 mg of anhydrous sodium acetate (Sigma-Aldrich) in 80 mL of water, adding 289 μL of glacial acetic acid (VWR Chemicals), adjusting the pH to 4.6 with HCl or NaOH if necessary and filling up to 100 mL with water. Other solvents were purchased as commercially available and used without further purification. Chemicals used for medium preparation were purchased from Carl-Roth GmbH.

### 2.2 Preparation of Aldehyde-agarose

Aldehyde-agarose was prepared based on the method published by Afanassiev.^33^

Agarose gels (1.5%, w/v) were prepared by dissolving agarose powder (300 mg) in water (20 mL) by heating in the microwave until boiling. NaIO_4_ (42.78 mg, final concentration of 10 mM, 0.20 mol) was added to the solution. The reaction mixture was heated until it was boiling in the microwave. The agarose was left to gel for 1 h at r.t. After that, the agarose gel was minced and washed with water (∼200 mL). Then, the gel was overlaid with water and frozen at −20 °C overnight. On the following day, the frozen gel was dried via lyophilization (Christ Alpha 1–4 LD, Vacuubrand RZ 2.5, > 50 °C, > 0.05 mbar) for a duration of 3 days using sample sizes of 50 mL.

Aldehyde-agarose samples were stored in 50 mL Falcon tubes sealed with parafilm in the dark at r.t.

### 2.3 Coupling of Aminooxy Dyes

For coupling of aminooxy dyes (either Aminooxy-5(6)-FAM or CF633 Dye aminooxy), an acetate buffer (10 mL) was added to aldehyde-agarose (150 mg). To the suspension, aniline (91.30 μL) was added, resulting in a 0.1 M anilinium-acetate buffer. After that, the respective aminooxy dye was added. The solution was stirred in the dark overnight at r.t. At the end of the reaction the suspended agarose was filtered and washed with water (∼100 mL), acetone (∼150 mL), and DMSO (5 mL). Then, the agarose was left for 90 min in DMSO (20 mL) for complete dissolution. Upon the addition of acetone, agarose precipitated and formed a dense solid structure. The precipitated agarose was filtered, washed with acetone (∼100 mL), and left in acetone (∼200 mL) overnight. The agarose was filtered again, washed with water (∼50 mL) and acetone (∼100 mL), and then left to dry in the air in the dark for 2 days.

Aminooxy dye-agarose samples were stored in 15 mL Falcon tubes sealed with parafilm in the dark at 4 °C.

#### Specific Procedure for Preparation of FAM-Agarose

5 mg of FAM-Aminooxy dye was dissolved in 5 mL of DMSO. From this stock solution, 0.5 mL (0.5 mg) was added to the reaction mixture.

#### Specific Procedure for Preparation of CF633-Agarose

1 mg of CF633-Aminooxy dye was dissolved in 10 mL of water. From this stock solution, 1 mL (0.1 mg dye) was added to the reaction mixture.

### 2.4 Rheometry

Rheometric measurements were conducted on a MCR301 Rheometer (Anton Paar) with a PP25 measuring geometry. Measurements were performed on 1.5% (w/v) agarose solutions in water with an amplitude gamma of 1% and a frequency of 1 Hz. Experiments were conducted while imposing a constant normal force (*F*_N_ = 0 ± 1 N). The lift position was set to 20.0 mm, and the measuring position to 0.6 mm. Before every measurement, the sample holder of the plate–plate geometry was preheated to 90 °C for 5 min. The agarose solution (250 μL) was then introduced in the gap of the plate–plate geometry, which was then brought to measuring distance. The outer ring of the geometry was filled with water, and the tempering chamber was shut down to prevent water evaporating from the agarose sample. The measurement was started after 5 min. The temperature was decreased in 3 intervals: (1) 90 to 61 °C at 1 K/15 s, (2) 60 to 50 °C at 0.5 K/15 s, and (3) 49 to 10 °C at 1 K/15 s. The final temperature was maintained at 10 °C for 15 min to ensure complete gelation. The gel point was determined by the crossover of temperature-dependent storage and loss modulus.

### 2.5 Fluorescence Microscopy

All fluorescence microscopy experiments were conducted on 1.5% (w/v) agarose hydrogel samples. The agarose samples were poured inside 24 well plates with glass bottom (Greiner Bio-One) using 500 μL of agarose per well. A confocal laser scanning microscope (cLSM) (Leica DMi8, Germany) was used. For conducting the experiments, the Leica Application Suite X (3.5.7.23225) software was used. Image data was saved in Leica Image File (lif) format. The data was further evaluated using ImageJ (1.53c).^34^

#### Fluorescence Recovery after Photobleaching

For fluorescence recovery after photobleaching (FRAP) measurements, 1 mL of water was added to the gelled agarose sample. The measurement was conducted using a 63×/1.20 WATER HC PL APO CS2 objective (Leica, Germany). Images were taken with a resolution of 0.10 μm/px, image size of 256 × 256 px, speed of 1800 Hz, and pinhole of 1.0 AU for FAM dye and CF633 dye samples. A 488 nm laser with an intensity of 0.1% or a 638 nm laser with an intensity of 5% was used for FAM and CF633 dye samples, respectively, if not stated otherwise. A PMT2 detector was used with a range of 504–650 nm and a SmartGain of 1000.3 V for FAM dye samples and a range of 648–750 nm and a SmartGain of 900 V for CF633 dye samples. A circular bleaching region was chosen with a diameter of 10 μm. At the start of the measurement, prebleaching images were recorded in 10 iterations (at intervals of 79 ms). Bleaching of samples was done in 100 iterations (at intervals of 79 ms) for agarose-bound FAM and 30 iterations (at intervals of 79 ms) for agarose-bound CF633 samples, if not stated otherwise. After the bleaching step, images were recorded in a first post bleaching step in 10 iterations (at intervals of 79 ms). Post-bleaching steps after this were chosen individually for the respective measurement. The measurements were limited to 500 s. Measurements were performed roughly 100 μm above the bottom of the glass slide of the well plate to avoid interface artifacts. Evaluation of FRAP measurements were done by Leica Application Suite X software and using Python packages pandas (v2.2.2) and matplotlib (v3.9.2). The fluorescence intensities of the bleaching area were normalized on the maximum intensity value and the intensity value directly after the bleaching of the respective measurement.

#### Fluorescence Intensity Measurements

For fluorescence intensity measurements, 2 mL of potassium phosphate buffer was added to the gelled agarose sample. Samples were introduced to different pH levels by increasing the pH of the buffer solution from pH 6 to 8 by adding 1 M NaOH or decreasing it from pH 8 to 6 by adding 1 M HCl. The amount of HCl or NaOH needed to achieve the desired pH values was determined beforehand (Supplementary Table S1). Measurements were conducted using a HC PL FLUOTAR 20*x*/0.55 DRY objective (Leica, Germany). For agarose-coupled FAM or aminooxy-FAM samples, images were taken with a resolution of 1.14 μm/px, image size of 512 × 512 px, speed of 400 Hz, and pinhole of 1.0 AU if not stated otherwise. A 488 nm laser with an intensity of 0.1% and a PMT2 detector with a range of 498–650 nm were used. For mixed samples with agarose-coupled FAM and agarose-coupled CF633 samples (1:1, w/w), images were taken with a resolution of 0.57 μm/px and image size of 1024 × 1024 px. A scanning speed of 400 Hz and a pinhole of 1.0 AU were used, if not stated otherwise. A 488 nm laser with an intensity of 0.1% and a PMT2 detector with a range of 498–628 nm were used to measure the FAM dye, and a 633 nm laser with an intensity of 0.2% and a HyD Smd3 detector with a range of 648–750 nm were used to measure the CF633 dye.

During the measurement, all images were taken at the same position as the *z*-stack with a *z*-interval of 2 μm over a total *z*-distance of 100 μm. Measurements were performed roughly 100 μm above the bottom of glass slide of the well plate to avoid interface artifacts. The mean intensity of all images was averaged, normalized on the maximum and minimum intensity, and plotted in dependence on correlated pH value.

### 2.6 Cell Culture

#### Bacterial Strains

The bacterial strains *Pseudomonas taiwanensi*s VLB120_eGFP^35^ and *Synechocystis* sp. PCC 6803 ^36^ used in this study were kindly provided by the Department of Solar Materials Biotechnology (SOMA) at the Helmholtz Centre for Environmental Research GmbH (UFZ).

#### Cultivation of *Synechocystis* sp. PCC 6803

*Synechocystis* sp. PCC 6803 was grown on BG11 agar plates, directly taken from cryo-stock, and incubated at 30 °C under 50 μmol photons m^–2^ s^–1^ (LED), ambient CO_2_, and 75% humidity for 1 week in a climatic chamber (Polyklima, Dinkelscherben, Germany). Precultures were then inoculated into 50 mL of YBG11 medium supplemented with 50 mM HEPES^37^ in a 250 mL baffled shake flask to an initial OD_7__5__0_ of 0.1. Main cultures were incubated under the same conditions on an orbital shaker (Celltron, Infors, Bottmingen, Switzerland) at 150 rpm (25 mm amplitude) for 4–7 days.

#### Cultivation of *Pseudomonas taiwanensis* VLB120_eGFP

*Pseudomonas taiwanensis* VLB120_eGFP was taken directly from cryo-stock and grown on 1.5% LB-Lennox plates at 30 °C overnight. Single colonies were inoculated into 20 mL of LB-Lennox medium with 100 μg/mL streptomycin in a 100 mL baffled shake flask and incubated at 150 rpm (25 mm amplitude) at 30 °C overnight. Precultures were then incubated for 18 h in 20 mL of M9 medium, adjusted to pH 7.2 using 10 M sodium hydroxide, containing (per liter) 5 g of glucose, 100 mg of streptomycin, 8.5 g of Na_2_HPO_4_·2H_2_O, 3 g of KH_2_PO_4_, 0.5 g of NaCl, and 1.0 g of NH_4_Cl. The following components were sterilized separately and added afterward (per liter of final medium): 2 mL of 1 M MgSO_4_ and 1 mL of trace element solution containing (per liter) 4.87 g of FeSO_4_·7H_2_O, 1.87 g of ZnSO_4_·7H_2_O, 0.15 g of CuCl_2_·2H_2_O, 1.5 g of MnCl_2_·4H_2_O, 4.12 g of CaCl_2_·6H_2_O, 0.25 g of Na_2_MoO_4_·2H_2_O, 0.3 g of H_3_BO_3_, 0.84 g of Na_2_EDTA·2H_2_O, and 82.81 mL of 37% fuming HCl.

### 2.7 Agarose Layer Preparation in Static Chip Systems

Pregrown cultures of *Synechocystis* sp. PCC 6803 and *Pseudomonas taiwanensis* VLB120_eGFP, as described above, were collected and washed twice with fresh medium without streptomycin after centrifugation for 2 min at 4000 × g. Bacterial solutions were then diluted to achieve specific cell densities ranging from 1 × 10^9^ to 5 × 10^9^ cells/mL and stored at 30 °C. A 1.5% w/v mix-gel agarose solution, consisting of a 1:1 ratio of FAM-agarose and LM-agarose, was prepared in ultrapure water by heating in the microwave until boiling, precooled, and stored at 35 °C. After that, the prepared agarose was added to the centrifuged cell pellets and resuspended.

For *Pseudomonas taiwanensis* VLB120_eGFP agarose layer preparation, 200 μL of the resuspended solution was pipetted onto the center of a glass ibidi μ-dish, forming a spot, and left to gel for 30 min. Subsequently, a 500 μL layer of 1.5% w/v mix-gel agarose solution was added on top and allowed to gel for 30 min.

For *Synechocystis* sp. PCC 6803 agarose layer preparation, 200 μL of the mix-gel agarose solution was pipetted onto the center of the ibidi μ-dish. Then, 200 μL of the bacteria-LM-agarose solution was added, surrounding the mix-gel spot. Finally, a 400 μL layer of 1.5% w/v LM-agarose solution was added on top. For each step, the agarose solution was allowed to gel for 30 min.

To compensate for bleaching and environmental effects on fluorescence intensity and pH changes during long incubation periods, a reference agarose layer was prepared under the same conditions as described above but without bacterial cells.

For both preparations, 4 mL of the respective culture medium with specific buffer capacity adjustments (M9: reduced Na_2_HPO_4_·2H_2_O and KH_2_PO_4_ to 1.2 and 0.43 g, respectively; YBG11: reduced HEPES to 1 mM) was added. Samples were then stored under their respective culture conditions, as described above, and analyzed either immediately after preparation or following overnight incubation in the dark for *Pseudomonas* and *Synechocystis*, respectively.

### 2.8 Fluorescence Intensity Measurements and Bacteria Visualization

Fluorescence intensity measurements of mix-agarose gels with embedded bacteria were conducted as described in Section 2.5 using samples prepared according to Section 2.7. Measurements were conducted using an HC PL APO CS2 40×/1.10 WATER objective (Leica, Germany). Images of mix-agarose gels containing a 1:1 ratio of FAM-agarose and LM-agarose with embedded *Synechocystis* sp. PCC 6803 was acquired at a resolution of 1.14 μm/px, image size of 256 × 256 px, scanning speed of 600 Hz in bidirectional mode, and pinhole size of 1.0 AU. Images of mix-agarose gels containing embedded *Pseudomonas taiwanensis* VLB120_eGFP were acquired at the same resolution, with an image size of 512 × 512 px, scanning speed of 600 Hz, and pinhole size of 1.0 AU. A 488 nm laser with an intensity of 0.1%, a SmartGain of 1000.3 V, and a PMT2 detector with a detection range of 500–580 nm was used for all measurements within the mix-agarose gels. To visualize *Pseudomonas taiwanensis*, VLB120_eGFP images were acquired using a 488 nm laser with an intensity of 2.0% and a HyD SMD 1 detector set to a detection range of 516–574 nm. This fits to the maximum excitation of the eGFP protein at 488 nm with its maximum emission being around 507 nm.^38^ *Synechocystis* sp. PCC 6803 was visualized using 633 nm laser with an intensity of 1.0%, a SmartGain of 631 V, and a PMT2 detector set to a detection range of 657–782 nm. With this, the autofluorescence of chlorophyll a and that of phycocyanin with a maximum excitation of around 625 nm can be captured in parallel.^39^

Prior to each cLSM imaging session, 1 mL of the supernatant medium was transferred under sterile conditions to a 1.5 mL Eppendorf tube. The pH was then measured using a pH electrode (InLab Micro Pro-ISM, Mettler Toledo). The electrode was calibrated before measurements on a daily basis. After measurement, the medium was transferred back to the sample.

To analyze a large volume of the agarose gel, 60 images were acquired within a 16-position squared tile set, sample centered, with a *z*-interval of ∼1.7 μm over a total depth of ∼100 μm. The mean intensity of all images was averaged, normalized to the mean intensity of the reference sample, and plotted as a function of the corresponding pH value, if not stated otherwise.

## 3 Results and Discussion

### 3.1 Covalent Coupling of Aminooxy Dyes to Agarose

Approaches for chemical coupling of functional molecules to agarose hydrogels, such as pH-sensitive dyes, have to consider several issues. First, subsequent labeling of agarose hydrogels after hydrogel formation and cell encapsulation has the drawback of unwanted harmful side-effects on living cells by reactive dye molecules and process conditions. On the other hand, modifying the agarose polymer chains prior to hydrogel formation requires careful consideration of the hydrogel formation process, as well as the role of the introduced functional groups and the possibly altered chain conformation on the gelling process. In this regard, hydrogen bond formation by hydroxyl groups and double helix formation between different chains are the major processes of gel formation in native agarose. Because of agarose’s chain constitution and its high polymer chain length, most of the hydroxyl groups are within the polymer chain with only a few on its chain ends at C3 of the β-D-galactose ring or C1 of the 3,6-anhydro-α-L-galactose ring, since they are used for the glycosidic bond (Figure 1). The secondary hydroxyl groups dominating the polymer backbone of agarose are considered poorly reactive due to steric hindrance.^11^ Furthermore, their significantly higher frequency, compared to hydroxyl groups on the chain ends, would likely lead to very high degrees of functionalization in such modification strategies and therefore strongly impact double helix formation and gelation due to the importance of hydroxyl groups in hydrogen bond formation.^11^

**Figure 1:**
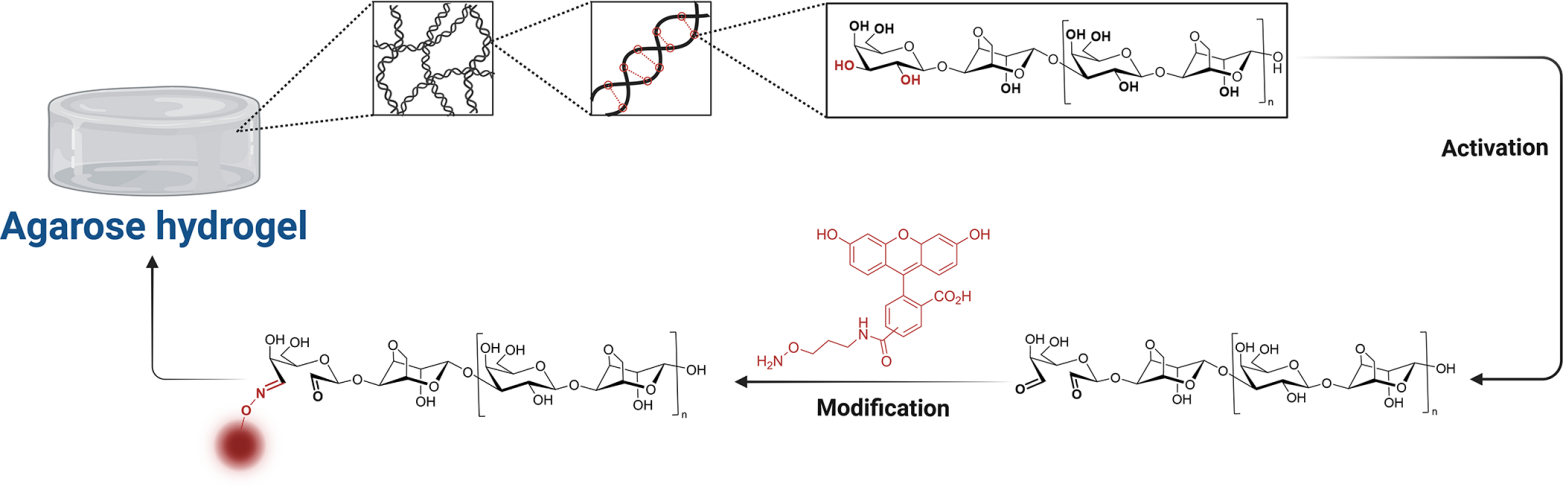
Approach of the dye coupling to agarose hydrogels. Overview of agarose chemical structure and origin of hydrogen bonds as basic mechanism for hydrogel formation together with a schematic depiction of the implemented synthesis strategy. The upper panel schematically shows the physical interaction of agarose chains, which are crucial to form the gel, as well as the chemical constitution of agarose with particular attention to the leading end of the polymer chain. The hydroxyl groups at the C3 and C4 positions of the β-D-galactose ring, which are important for the reported synthesis strategy, are highlighted in red. In the lower panel, the agarose polymer chains are shown, after the activating step, generating the aldehyde groups, and the modification step, coupling the dye molecule. Alternatively, like depicted for the aminooxy-5(6)-FAM molecule, the CF633 dye was immobilized in the same manner.

To modify agarose polymer chains prior to hydrogel formation and to alter agarose properties as little as possible but still allow for coupling functional molecules, we used an end-on modification strategy for chemical modification of agarose polymer chains. In a two-step process, we activated end-on hydroxyl groups of agarose at the C3 and C5 positions of the β-D-galactose ring and subsequently coupled the molecule of interest to the activated aldehyde moieties as depicted in Figure 1.

To the best of our knowledge, using an end-on modification strategy to couple FAM dye molecules to agarose has not been performed so far, as typically side-on strategies are used for functionalizing agarose. In contrast to the often-used epoxy-activation^40^ of agarose, we chose an aldehyde activation to specifically modify the C3 and C5 position of the β-D-galactose ring on the chain end of the agarose polymer chain. This was made possible by reacting agarose with sodium periodate for 1 h after boiling in a microwave. Sodium periodate is used for the oxidative cleavage of vicinal diols, which leads, in this case, to a formation of two aldehyde groups at the chain ends of the agarose.^41^ Since the number of these vicinal diols at the chain end of agarose is very limited, good control of the degree of modification can be achieved by simply choosing reaction conditions for a complete conversion of these end points. This is in contrast to the often-used side-on strategies, where performing low degrees of modification is usually harder to realize. Through this procedure, a very low degree of modification could be achieved, reducing the impact of the disturbance of the native agarose gel properties. Coupling of aminooxy dyes was performed in 0.1 M anilinium-acetate buffer, as it has been proven to significantly increase the reaction rate of the oxime ligation through nucleophilic catalysis.^42,43^ During the washing procedure of the reaction product, DMSO was used, which is a solvent that dissolves agarose regardless of temperature by inhibiting interchain hydrogen bond and double helix formation.^20,44^ A subsequent addition of acetone led to the formation of dense, solid agarose chunks, which were washed further (see Section 2.3). For synthesis of pH-sensitive agarose hydrogels, aminooxy-FAM was chosen for the reaction, as FAM is known to exhibit pH-dependent fluorescence in the range of 6 to 8, which is a relevant range of the microbial cell studies.^26^ Furthermore, aminooxy-CF633 dye was used in the agarose modification to synthesize CF633-agarose exhibiting no dependence of fluorescence characteristic on pH and serving as reference samples in respective experiments.^45^

### 3.2 Rheometric Analysis of Modified Agarose

To analyze the elastic properties of native and aldehyde-activated agarose hydrogels, rheometric measurements were performed. This was done with the aim to provide evidence for a neglectable influence of agarose modification on gel formation, proving the end-on activation of agarose chains with low numbers of affected hydroxyl groups. As previously described, a side-on aldehyde functionalization has a drastic effect on gel mechanics, by strongly reducing rigidity.^46^ Furthermore, modifications like carboxylation of the agarose backbone leads to softer gels with lower gelation temperatures in comparison to native agarose.^17,22^

Traces of sulfate groups present on the agarose chain may modify gel properties, which is why all samples used have sulfate contents smaller than 0.15%, as given by the supplier. Therefore, their possible effect is neglected hereafter.

For agarose samples, a decrease in elastic modulus, due to network loosening and volume contraction, as a result of water loss, has been reported.^47,48^ For this reason, we specifically took care to inhibit water evaporation during rheometric measurements (see Section 2.4). Our results show the expected behavior with minor changes of elastic properties by aldehyde activation of agarose (Figure 2). Native agarose samples show a typical gelling behavior and elastic properties (Figure 2A). A gel point of 35 °C was determined and storage modulus reached values around 25 kPa at 10 °C, which is in line with other rheological measurements of agarose hydrogels indicating storage moduli in the range of some kPa.^49–52^ Aldehyde-activated agarose (Figure 2B) showed a similar behavior with a slightly lower gel point of 34 °C and storage modulus of around 20 kPa. The slightly lower gel point and lower storage modulus can be explained by the disturbance of hydrogen bonding between the agarose chains upon aldehyde group generation at end-on hydroxyl groups. This is in accordance with the literature on a similar functionalization with carboxylic groups.^17,22^ However, while side-on modifications, such as carboxy-functionalization, show stronger disruptions of gel mechanics, our end-on approach results in agarose gels with nearly unaltered rheological properties.

**Figure 2:**
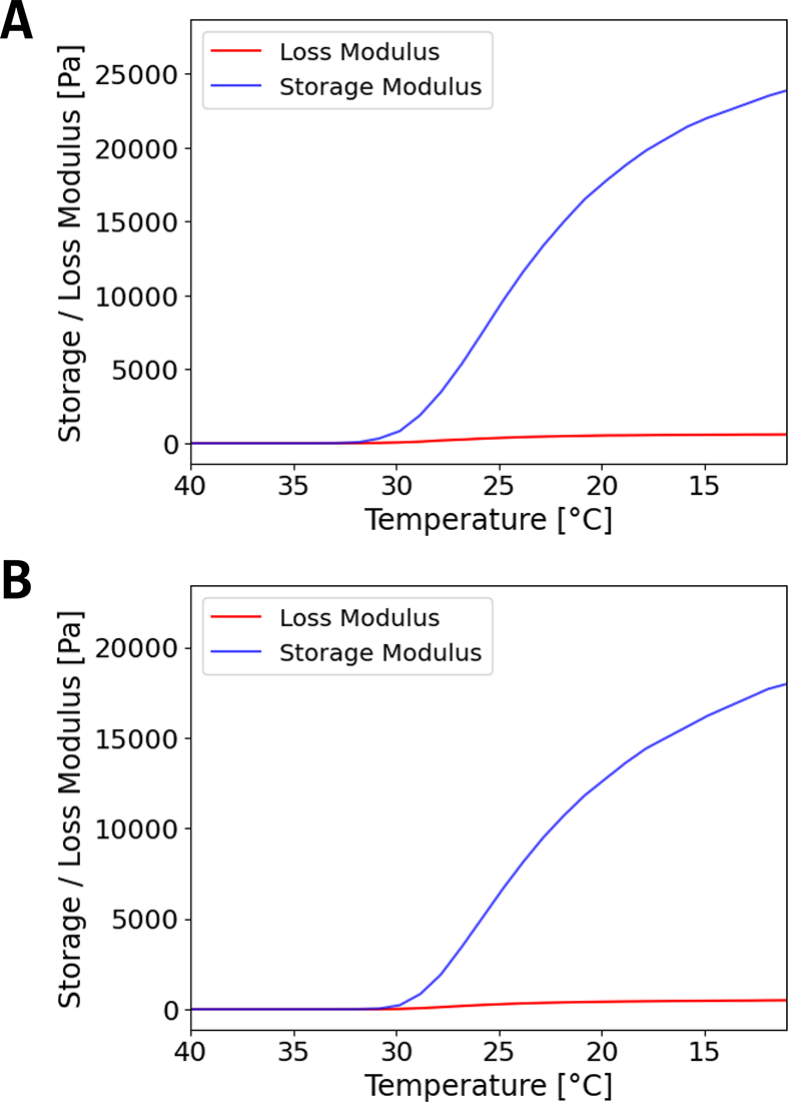
Determination of the elastic modulus of agarose hydrogels with and without end-on aldehyde activation. Rheometric measurement of agarose (A) and aldehyde-activated agarose (B). Depicted are representative measurements of loss modulus and storage modulus of agarose samples plotted against the temperature, measured at 1 Hz frequency and 1% strain amplitude. The gel point was determined by the crossover of temperature-dependent storage and loss modulus with 35 °C for the agarose sample (A) and 34 °C for the aldehyde-agarose sample (B).

In our rheological measurements, we also considered volume variations during sol–gel transition. Most of the rheological experiments on the gelation of thermoreversible gels reported in the literature have been performed with a constant gap setup and therefore do not account for the contraction of the gel during gelation. This however leads to falsified results and a drift to ever larger values of moduli after gelation is over. Because of this, a normal-force-controlled procedure was used by imposing a constant normal force equal to zero during the measurement. This method has been proven as more reliable in measuring linear properties and gelation dynamics of thermoreversible gels.^53^

Therefore, we checked at the end of the experiment that storage modulus reaches a plateau at the end of gelation (Supplementary Figure S1). Our time-dependent measurements, up to 15 min after reaching the final temperature of 10 °C, indicate a plateau for the storage modulus, underlining the precision of our normal force-controlled procedure.

### 3.3 Proof of Covalent Coupling of Aminooxy Dyes to Agarose using FRAP

FRAP was used to confirm the covalent coupling of aminooxy dyes to the end-on aldehyde-activated agarose polymer chain. Although there are several studies on optical sensors using agarose as scaffold, to our knowledge, in-depth analysis of the covalent coupling of agarose-coupled dyes has not been performed so far.^40,54,55^ However, the proof of removal of unbound dye is crucial for making a statement on the reliability in sensor application. Noncovalently bound and slowly released dye molecules can disturb position-dependent measurements of pH inside agarose hydrogel samples, and freely diffusing dyes can influence behavior of living cells in *in vitro* experiments. Therefore, we investigated in detail the washing durations and solvent conditions and proved stable, covalent coupling of dyes to agarose by FRAP in the hydrogel samples.

The FRAP measurements in Figure 3 demonstrate the different removal efficacy of unbound FAM dye molecules by different washing techniques. The FRAP measurements depict the insufficient washing of the agarose-dye sample in the first approach (Figure 3A, FAM short water short). Here, the FAM-agarose sample was shortly washed with 150 mL of water and then left in 300 mL of water over 12 days with 2 times water exchange, each after 3 days, before a final filtration and washing step with 150 mL of water. The FRAP curve shows a strong recovery (51%) after bleaching (and even noncomplete bleaching), attributed to a high amount of free unbound dye inside the sample. However, already here, an immobile part (49%) was observed, indicating covalent coupling. An additional dialysis step of the same sample in 300 mL of Milli-Q with a water exchange after 3 days and after 4 days still showed the presence of unbound FAM molecules, although the amount was substantially decreased (20% recovery after 3678 s, Supplementary Figure S2, Figure 3, FAM water long). As the efficacy of removal is of course dependent on the size of the respective sample and the respective diffusion rates, washing with water of smaller samples has also been tested (Supplementary Figure S3). Here, circular samples with a diameter of 13 mm and a thickness of around 100 μm were used. Although substantial amounts of free dye could be removed, samples were difficult to handle and clean up still required substantial timeframes of multiple days. Therefore, a washing protocol involving acetone was established, which uses acetone washing overnight as the main step. Acetone washing of dye-coupled agarose samples showed no FRAP recovery, proving the absence of freely moving FAM dye molecules and a complete covalent coupling of the dye to the agarose hydrogel (Figure 3A, FAM acetone). We also checked much longer FRAP recovery times up to 120 min at which no recovery was observed, too. This measurement further supported the covalent coupling of aminooxy-FAM to aldehyde-activated agarose (Supplementary Figure S2).

**Figure 3:**
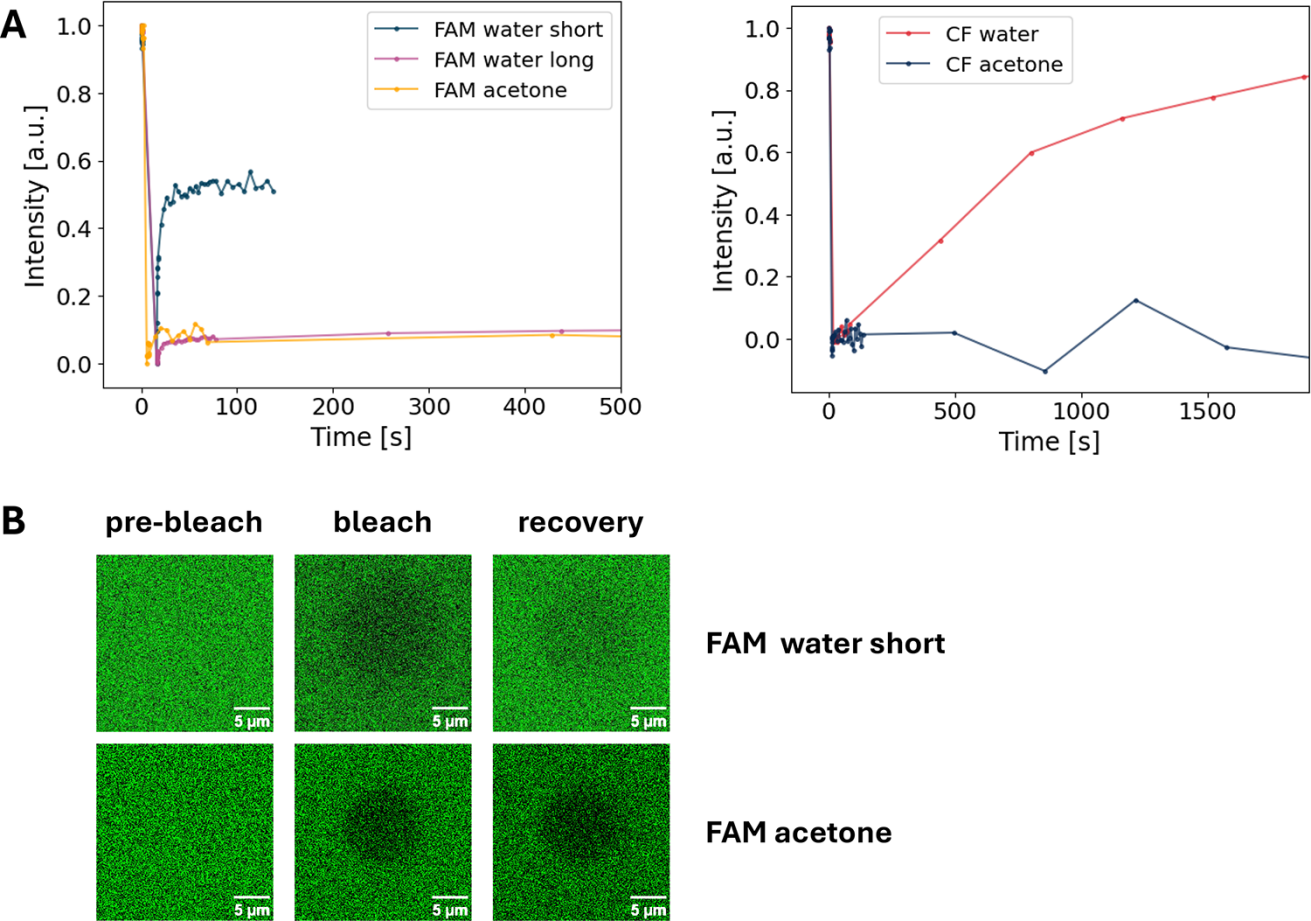
Determination of covalent coupling of dyes to agarose hydrogels using FRAP. Depicted are different representative single spot measurements of agarose-coupled dye samples during FRAP analysis (A). Three agarose-coupled FAM samples are shown representing different washing procedures of the samples including washing procedures solely with water for varying time durations of washing (FAM-water, short, 12 days; FAM-water, long, 21 days (measured with 1.5% laser intensity)) and the finalized procedure including washing steps with DMSO and acetone (FAM acetone, bleaching was done in 10 iterations). Furthermore, two agarose-coupled CF633 samples are shown, representing a washing procedure solely with water (CF-water, bleaching was done in 50 iterations) and the finalized washing procedure (CF-acetone). For each measurement, fluorescence intensity values were normalized on the maximum intensity value and the intensity value directly after bleaching. Representative cLSM images are shown (B) at the beginning of the FRAP measurement (prebleach), directly after the bleaching step (bleach), and at the end of the measurement (recovery) for two different sample types showing high recovery (FAM water short) and no recovery (FAM acetone) after bleaching.

FRAP measurements were also performed to verify covalent coupling and sufficient washing of agarose samples after coupling aminooxy-CF633 dyes. The CF633 dye is known to exhibit a fluorescence characteristic independent of pH and were indented to be used as reference dye in agarose hydrogels.^45^ These measurements again showed the insufficient washing of agarose-dye sample in water (96% recovery) but sufficient washing and covalent coupling using washing in acetone (Figure 3A, CF water, CF acetone). Herein, the initial washing procedure (water) included subsequent washing steps with 50 mL of water, 50 mL of acetone, and 50 mL of water before leaving the sample overnight in 200 mL of water, and a final washing with 50 mL of acetone and 50 mL of water. Although the CF633 dye is described as highly water-soluble,^45^ this washing procedure achieved no complete removal of unbound dye out of the hydrogel. Only the final procedure with washing and keeping the sample in acetone overnight allowed a complete removal of free dye, similar to the FAM-agarose samples. This result is indicated by the absence of any recovery in the FRAP measurements (Figure 3A, CF acetone).

These experiments showed that extensive washing steps are needed to fully remove any noncovalently bound dye molecules. Even though the dye molecules themselves are water-soluble, washing with organic solvents was crucial for successful removal of unbound dye.

### 3.4 Test of pH Responsiveness of Agarose-Coupled Dyes

cLSM was used to show the pH-dependent fluorescence intensity of the agarose-coupled aminooxy-FAM dye. A pH dependency was previously shown for the free^26^ as well as for oligonucleotide-bound FAM.^27–29^

We first compared the behavior of free aminooxy-FAM dye to that of agarose-coupled FAM dye samples (Figure 4A). As shown, agarose-coupled FAM dye samples possess similar pH dependence to their respective free dye samples. The change of intensity does not follow a linear trend but an expected monotonic decrease in fluorescence intensity with decreasing pH, in the range of pH 6 to pH 8, which is in accordance with literature reports.^27,31^ This trend is independent of the direction of the pH change, as both a pH change from 6 to 8, as well as a change from 8 to 6 showed the same characteristic.

**Figure 4:**
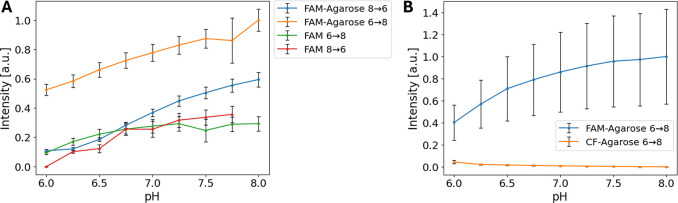
pH-dependent fluorescence intensity measurements of free dyes and agarose-coupled dyes in the hydrogels. (A) Representative measurements of free aminooxy-FAM and agarose-coupled FAM in hydrogels. (B) Representative measurements of agarose-coupled FAM in comparison to agarose-coupled CF633 in hydrogels. Samples were subsequently kept at different pH values (from pH 6 to 8 (6→8) and from pH 8 to 6 (8→6)) and fluorescence intensity was measured by cLSM. Normalized mean values ± SD of z-stack cLSM images are plotted. Intensity values are normalized on the maximum and minimum intensity value of the respective measurement series.

Furthermore, a mixed sample of FAM-agarose and CF633-agarose was prepared to show a proof-of-concept to have an internal pH-independent fluorescence reference included in the agarose hydrogel to account for depth and scattering dependent changes in fluorescence intensity. CF633 is known to have pH-independent fluorescence characteristics in the relevant pH range. Our results show that the designed system meets the expectations, as the CF633-agarose intensity showed almost no changes with pH, while the FAM-agarose signal showed the similar behavior like in the preceding experiments, with an increase in fluorescence intensity with increasing pH (Figure 4B).

Hence, covalent coupling of aminooxy dyes (FAM and CF633) to aldehyde-activated agarose showed their successful usage as pH indicators within agarose hydrogels. Their response time of 3 min is within a reasonable range, considerably faster than that of some nanoparticle sensors.^56^

### 3.5 Determination of Metabolic Activity of Microbial Cells in Agarose Hydrogels by pH Measurements

To confirm the applicability of the newly prepared pH sensor for measuring metabolic activities of incorporated microbial cells, a proof of principle study with *Pseudomonas taiwanensis* VLB120_eGFP was performed. Samples were prepared according to Section 2.7 (see also Figure 5, left). To adjust the gelling temperature of the agarose-coupled FAM to a suitable range for cell incorporation, a 1:1 mixture with LM-agarose was used for sample preparation. Pre-experiments confirmed the viability and further growth of cells within the agarose hydrogels after gelling (Supplementary Figure S5). Measurement of the fluorescence intensity was performed on the agarose-coupled-FAM mixed with LM-agarose surrounding the inner agarose matrix, embedding the cells.

**Figure 5:**
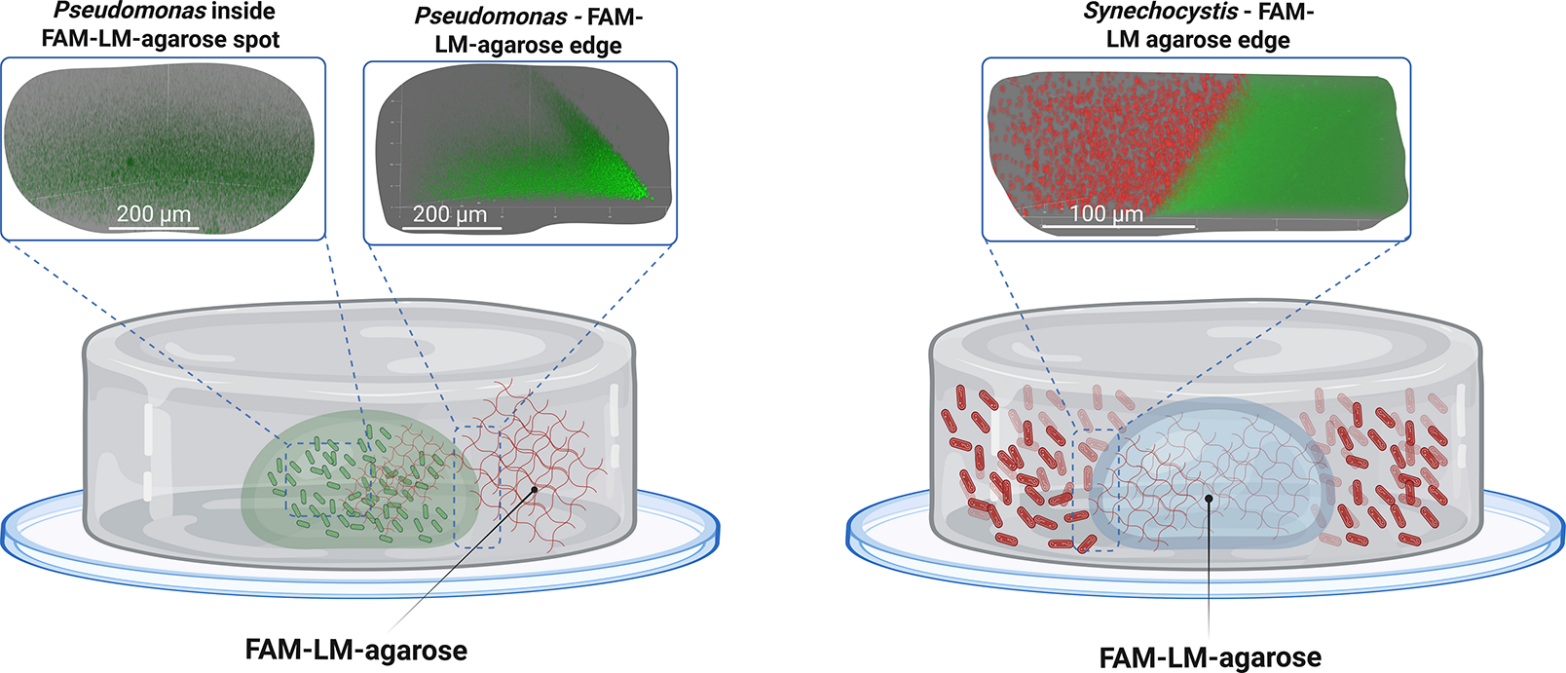
Schematic representation of the setup to measure the metabolic activity of microbial cells using agarose-coupled FAM. The illustration shows sample preparations for *Pseudomonas taiwanensis* VLB120_eGFP (left) and *Synechocystis* sp. PCC 6803 (right). Agarose-coupled FAM were mixed with low-melt agarose (FAM-LM-agarose) to achieve a mix-agarose with lower gelling temperature. Representative cLSM images (top row) depict *Pseudomonas taiwanensis* cells located within the pH sensor spot and at its edge (left), and *Synechocystis* cells embedded in low-melt agarose adjacent to the edge of the pH sensor spot (right).

As a strictly aerobic bacterium, *Pseudomonas taiwanensis* VLB120_eGFP consumes carbon sources during growth, producing organic acids and CO_2_ as a byproduct of cellular respiration and growth-related metabolism. In aqueous conditions, the released CO_2_ can form carbonic acid. The carbonic acid formed this way, together with the organic acids produced, lead to a decrease in pH in an active metabolic state.^57,58^

As shown in Figure 6, our measurements meet this expectation of a decreasing pH with an increasing metabolic activity and duration. After 24 h of incubation, the strong change in fluorescence intensity of the *in situ* pH sensor and the externally measured pH are clearly correlated.

**Figure 6:**
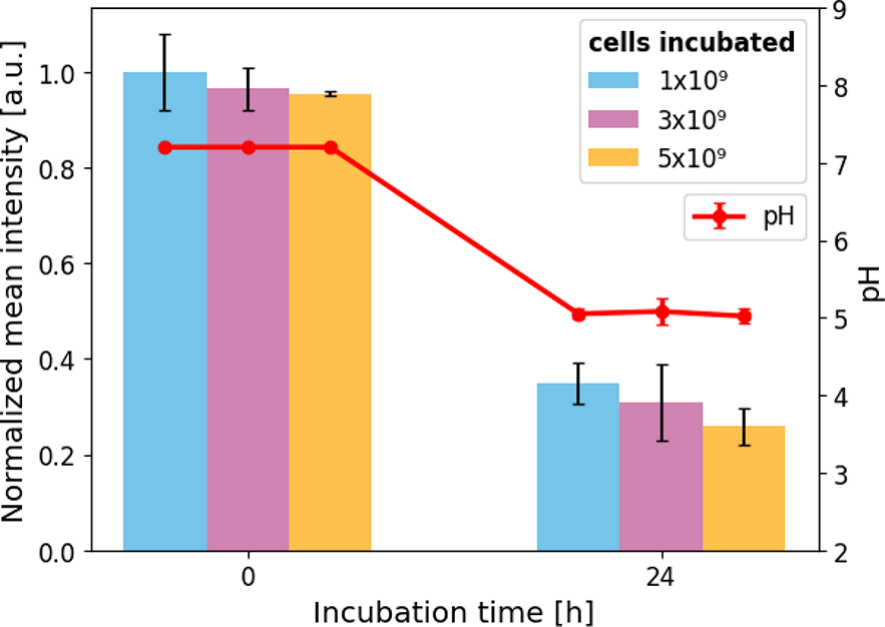
pH-dependent fluorescence intensity measurements of agarose-coupled FAM with incorporated *Pseudomonas taiwanensis* VLB120_eGFP cells. Measurement of the fluorescence intensity of agarose-coupled FAM is dependent on the amount of *Pseudomonas taiwanensis* VLB120_eGFP cells incorporated into the gel matrix. Normalized mean values ± SD of *z*-stack cLSM images are plotted. In addition to each measurement, pH values of the samples were recorded using a pH electrode.

To extend this proof of principle study, another measurement was performed using phototrophic *Synechocystis* sp. PCC 6803 cells (Figure 5, right). In this setup, the cells were embedded in a LM-agarose matrix surrounding the pH sensor composed of agarose-coupled FAM mixed with LM-agarose. This specific arrangement of a cell-free pH sensor spot was chosen to avoid measurement errors and intensity distortions caused by light absorption and scattering from the cells while still capturing environmental changes resulting from cell metabolism.

Oxygenic photosynthesis is carried out by microalgae or cyanobacteria such as *Synechocystis* sp. PCC 6803, which can increase pH through CO_2_ fixation or proton uptake, resulting in a shift of the carbonate equilibrium.^59^ Hence, we would expect an increase in pH and fluorescence intensity of the in situ pH sensor, in contrast to the preceding study using *Pseudomonas taiwanensis*.

Our results show that an increase in externally measured pH was observed with increasing incubation time, with the highest shifts occurring at the highest cell densities, as expected (Figure 7B). *In situ* fluorescence intensity measurements of the cell-free pH sensor spot indicate a correlation to these measurements (Figure 7A), with intensity values increasing as the pH rises for incubation times up to 24 h for all cell densities. Furthermore, the experiment suggests that higher cell densities result in a steeper increase in pH and a higher fluorescence intensity of the pH sensor compared to lower cell densities. For an incubation time of 2 and 3 days the dependence of the pH and fluorescence intensity on cell density is still visible. However, an overall decrease in fluorescence intensity was observed. This is accounted for a photobleaching of the agarose-coupled FAM dye due to the long incubation under constant light to cultivate phototroph cyanobacteria. In these long-term measurements, the absolute intensity decreased to very low values, which were not really suitable anymore for a reliable measurement (Supplementary Figure S4).

**Figure 7:**
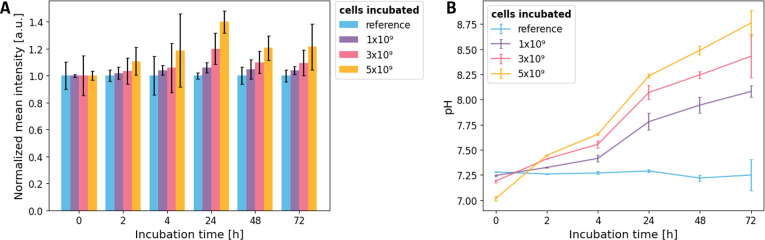
pH-dependent fluorescence intensity measurements of agarose-coupled FAM with the incorporated *Synechocystis* sp. PCC 6803 cells. (A) Measurement of the fluorescence intensity of agarose-coupled FAM is dependent on the amount of *Synechocystis* sp. PCC 6803 cells incorporated into the gel matrix. Mean intensities were normalized to the respective value at *t* = 0 h of each measurement to improve the visual representation of changes within each sample type. Normalized mean values ± SD of *z*-stack cLSM images are plotted. (B) In addition to each measurement, pH values of the samples were recorded using a pH electrode.

## 4 Conclusions

In this study, we successfully synthesized dye-functionalized agarose. FAM and CF633 dyes were covalently coupled to agarose polymers to serve as a fluorescence pH sensor (FAM) with an internal reference (CF633). This system was applied to monitor the pH value changes, in the relevant range of pH 6 to 8, as an indicator for metabolic activity of encapsulated microbial consortia of chemoheterotroph and phototroph strains.

The end-on modification of agarose using aldehyde activation was proven to be an efficient approach to avoid severe disturbances of the agarose gel formation ability and elastic properties of resulting hydrogels, which were measured via rheometric analysis, showing a storage modulus of around 20 kPa at 10 °C as is in the usual range for 1.5% agarose gels. Efficient washing procedures and FRAP experiments confirmed the covalent coupling of the dyes, visible due to the lack of a recovery in the recorded FRAP spectra as opposed to a recovery of 51% for samples that were simply washed with water. Varying culturing conditions for microbial consortia, using *Pseudomonas taiwanensis* VLB120_eGFP and *Synechocystis* sp. PCC 6803, within the agarose hydrogels demonstrated the system’s applicability for investigating the metabolic activity of different biomimetic biofilm systems. Direct coupling of the sensor molecule to the agarose chain overcomes common disadvantages of nanoparticle sensors like nonuniform particle sizes, aggregation, inhomogeneous dispersion, and a tendency to be endocytosed into cells.^60,61^ Furthermore, pH can be directly measured in the whole gel matrix without incorporation of particles or electrodes, which could disrupt the scaffold.

Our study presents a new tool of an agarose modification strategy to end-on couple aminooxy-containing functional molecules to agarose polymer chains without altering agarose gel mechanics. Using this strategy, we could prepare a new scaffold type for studying microbial interactions through a fast optical pH readout, with the option to achieve spatially resolved readouts. The approach allows for the integration of dyes sensitive to different pH ranges or dyes with higher photostability, too.^40,62^ Moreover, other functional molecules could be coupled using this strategy, for example, enzymes like proteases.^63^ With this, preparation of similar biomimetic biofilm scaffolds for analyzing bacterial behavior can be envisioned, advancing the field of biofilm and microbial research.

## Supplementary Material



## Data Availability

Data supporting the findings of this study have been included as part of the Supporting Information. Detailed raw data are available from the corresponding author upon reasonable request.

## Notes

pH titration tables, time-dependent rheometric analysis, FRAP spectra, non-normalized and nonreferenced metabolic activity (via pH) measurements (PDF)
