# Supplementary material for: Coupling pH-Responsive Dyes to Agarose Hydrogels for Monitoring Metabolic States of Encapsulated Phototrophic Microbial Consortia
Source: ACS Appl Bio Mater. 2025 Nov 12;8(12):10855–65. doi: 10.1021/acsabm.5c01556 (PMC12709568; doi:10.1021/acsabm.5c01556)
Supplement: Supplementary file 1 [file mt5c01556_si_001.pdf]

Supporting Information

Coupling pH-responsive dyes to agarose hydrogels for monitoring metabolic states of encapsulated phototrophic microbial consortia

*Matthias Ueberham, Christian Danneberg, Lisa-Maria Wagner and Tilo Pompe**

Institute of Biochemistry, Leipzig University, 04103 Leipzig, Germany.

*Email: tilo.pompe@uni-leipzig.de

1. Extended Experimental Part

1.1 pH titration of buffer solution

For pH dependent experiments, buffer solutions with a pH of 6.00 and 8.00 have been prepared. For the buffer solution with a pH of 6.00, K_2HPO_4 (120.25 mg) and KH_2PO_4 (586.50 mg) were dissolved in40 mL water. The pH was adjusted to 6.00 using HCl or NaOH if necessary, before filling up the buffer solution to 50 mL. The buffer solution with a pH of 8.00 was prepared in the same manner using K_2HPO_4 (814.09 mg) and KH_2PO_4 (44.39 mg).
To test the amount of NaOH and HCl necessary to adjust the pH of the two buffer solutions to the desired value, titration experiments have been performed twice using 2 mL of the respective buffer solution. 1 M HCl or 1 M NaOH was pipetted into the buffer solution. The solution was left for at least 3 min until equilibrium was reached and then the pH value was noted. pH values were recorded with a InLab Micro Pro-ISM pH electrode (Mettler Toledo). Relevant values for the adjusted pH values during pH dependent experiments are given in Table S1.

Table S1: Titration of phosphate buffers to determine the amount of HCl and NaOH needed to adjust pH values.

pH 6.00 buffer		pH 8.00 buffer	
**pH**	**1 M NaOH [µl]**	**pH**	**1 M HCl [µl]**
6.00	0	8.00	0
6.25	12	7.75	11
6.50	34	7.50	24
6.75	60	7.25	44
7.00	90	7.00	69
7.25	116	6.75	96
7.50	137	6.50	123
7.75	153	6.25	143
8.00	163	6.00	159

2. Extended Results & Discussion

2.1 Time-dependent rheometric analysis of modified agarose

To check whether the storage modulus reaches a plateau at the end of gelation, rheometric measurements have been performed and storage and loss modulus of the samples were plotted against time. For that, after reaching the final temperature of 10 °C, the temperature was kept at 10 °C for up to 15 min, with additional measurements at each minute.

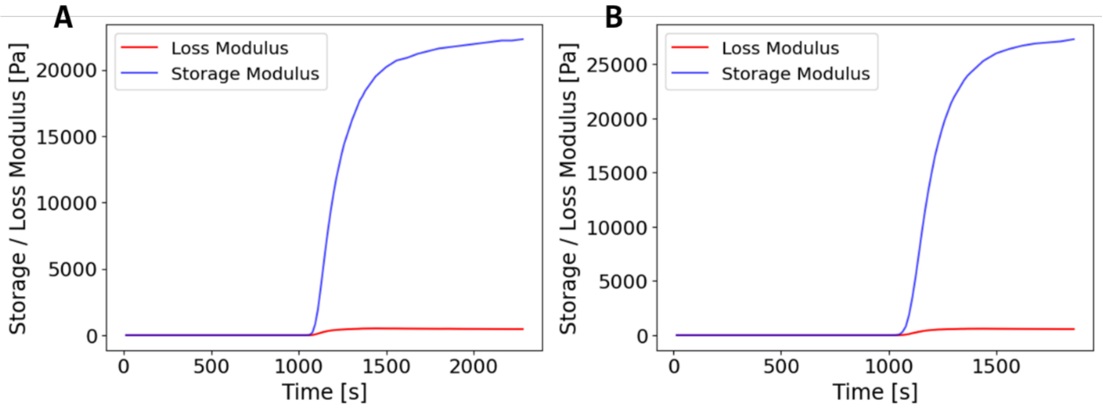

Figure S1: Determination of storage and loss modulus of agarose hydrogels with and without end-on aldehyde activation over time. Rheometric measurement of aldehyde-activated agarose **(A)** and agarose **(B)**. Depicted are representative measurements of loss modulus and storage modulus of agarose samples plotted against the time, measured at 1 Hz frequency and 1% strain amplitude.

2.2 Extended proof of covalent coupling of aminooxy-dyes to agarose using FRAP

FRAP measurements over very long time periods were performed to check whether a slow diffusion of free dye can be found. Measurements involving samples cleaned with the final washing procedure (Figure S2, acetone) showed no recovery and further supported the statement that the FAM-dye was covalently coupled to the agarose scaffold. This is particularly visible when comparing two samples washed solely with water even for very long times of measurements up to 4000 s (Figure S2, water long). Decreases in intensity over the measurement interval are an effect of the bleaching of the FAM dye during intensity postbleach measurements, due to its low photostability.

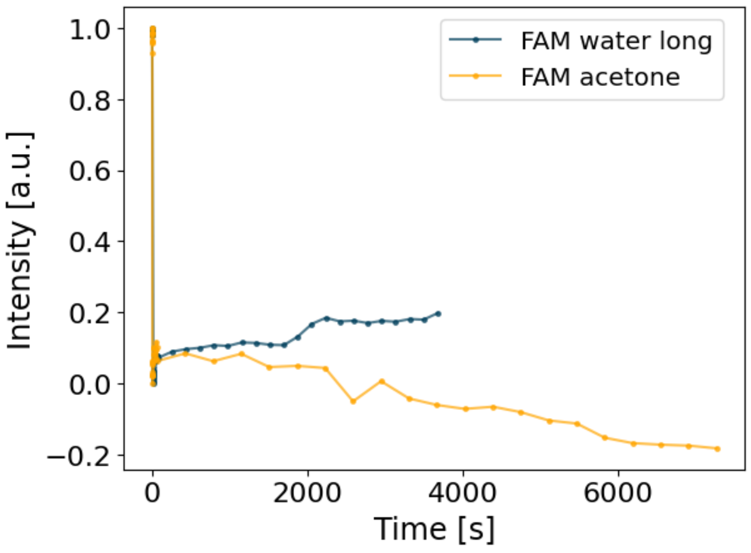

Figure S2: Determination of covalent coupling of FAM-dye to agarose hydrogels using FRAP in a long-time measurement. Depicted are agarose-coupled FAM dye sample during FRAP analysis. One sample was prepared using the finalized washing procedure including washing steps with DMSO and acetone (FAM acetone, bleaching was done in 10 iterations) and the other sample was prepared using a washing procedure with water and a dialysis in water over 21 days with four times water exchange (FAM water long). Fluorescence intensity values were normalized on the maximum intensity value and the intensity value of the first measurement point after bleaching.

FRAP measurements of small-size samples have been performed, too, to check whether a more efficient diffusion of free dye during washing with water occurs because of the small sample size. Agarose samples were prepared on coverslips with a diameter of 13 mm which were pre-cleaned for 10 min with EtOH in an ultrasonic bath and dried with nitrogen. Agarose was dissolved in a microwave and held at 55 °C in a water bath. Per coverslip 15 µm of 1.5% agarose-coupled dye was pipetted and a second coverslip was placed on top. The agarose was left to gel (6 min for agarose, 4 min for agarose-coupled FAM). Afterwards the second coverslip was cautiously removed and the sample was left to gel for another 3 min before placing it in a beaker filled with Milli-Q water. Dialysis in water was carried out for four days with two times water exchange after 20 h and 48 h. FRAP measurements involving samples cleaned with this procedure showed no recovery, indicating the successful removal of free FAM dye molecules.

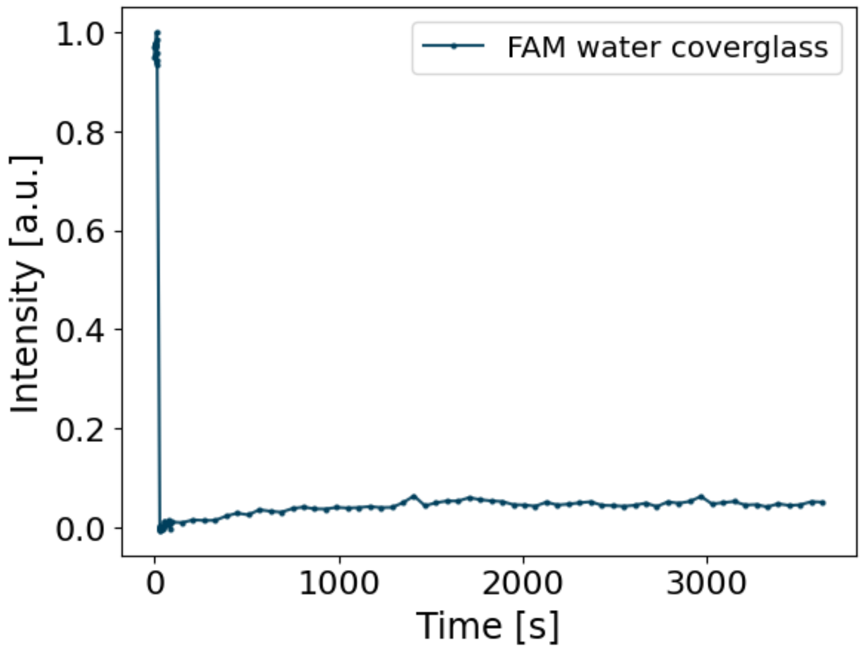

Figure S3: Determination of covalent coupling of FAM-dye to agarose hydrogels using FRAP with samples prepared on coverslips. Depicted is an agarose-coupled FAM dye sample during FRAP analysis. The circular sample with a diameter of 13 mm and a thickness of around 100 µm was prepared using the washing procedure with water and a dialysis in water over 4 days with two times water exchange. Fluorescence intensity values were normalized on the maximum intensity value and the intensity value of the first measurement point after bleaching.

2.2 Extended determination of metabolic activity of microbial cells in agarose hydrogels by pH measurements

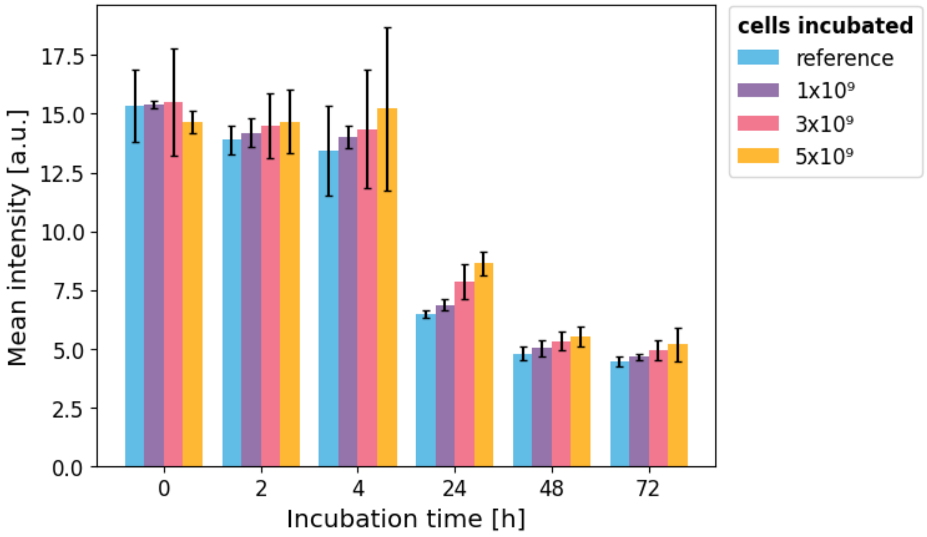

Figure S4: Non-referenced and non-normalized pH-dependent fluorescence intensity measurements of agarose-coupled FAM with incorporated *Synechocystis* sp. PCC 6803 cells.

2.3 Cell growth experiments

Growth experiments using *Pseudomonas taiwanensis* VLB120_eGFP in either low-melt agarose and native agarose (1:1, Figure S5, lm-ag-bact) or low-melt agarose and FAM-agarose (1:1, Figure S5, lm-fam-bact) were conducted using a Tecan Reader (600 nm). Agarose samples were prepared as described in section 2.7. Cultivated *Pseudomonas taiwanensis* VLB120_eGFP as well as the medium used for all samples was prepared as described in section 2.6. All samples were referenced to a blank sample containing only FAM-agarose mixed with low-melt agarose (1:1) without cells. The experiments show a similar growth behavior in agarose gels containing native agarose and agarose gels containing FAM-functionalized agarose. This finding indicates a non-cytotoxic character of the prepared pH-sensor gels.

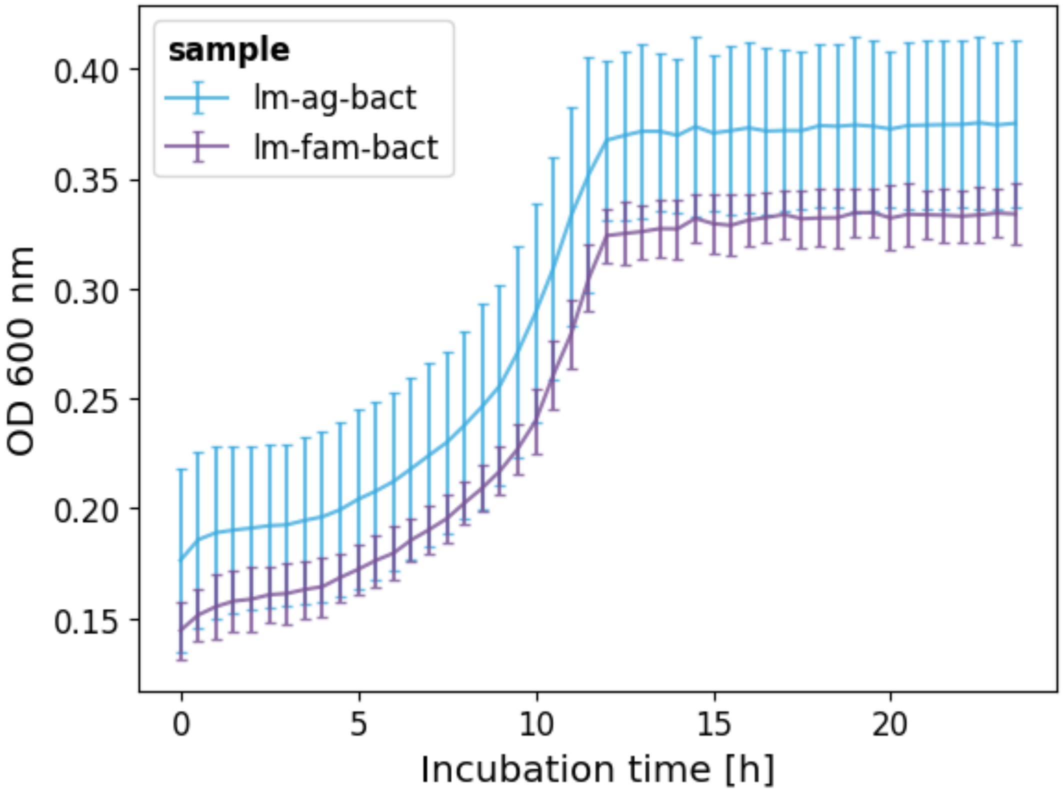

Figure S5: Growth curves of *Pseudomonas taiwanensis* VLB120_eGFP in low-melt agarose -agarose mix (lm-ag-bact) or low-melt agarose – FAM-agarose mix (lm-fam-bact) samples.
